# UHRF1 Promotes Proliferation of Human Adipose-Derived Stem Cells and Suppresses Adipogenesis via Inhibiting Peroxisome Proliferator-Activated Receptor *γ*

**DOI:** 10.1155/2019/9456847

**Published:** 2019-07-22

**Authors:** Ke Chen, Zi Guo, Yufang Luo, Jingjing Yuan, Zhaohui Mo

**Affiliations:** Department of Endocrinology, Third Xiangya Hospital of Central South University, Changsha, Hunan 410013, China

## Abstract

Once the adipose tissue is enlarged for the purpose of saving excess energy intake, obesity may be observed. Ubiquitin-like with PHD and RING Finger domains 1 (UHRF1) is helpful in repairing damaged DNA as it increases the resistance of cancer cells against cytocidal drugs. Peroxisome proliferator-activated receptor *γ* (PPAR*γ*), an important nucleus transcription factor participating in adipogenesis, has been extensively reported. To date, no study has indicated whether UHRF1 can regulate proliferation and differentiation of human adipose-derived stem cells (hADSCs). Hence, this study aimed to utilize overexpression or downregulation of UHRF1 to explore the possible mechanism of proliferation and differentiation of hADSCs. We here used lentivirus, containing UHRF1 (LV-UHRF1) and siRNA-UHRF1 to transfect hADSCs, on which Cell Counting Kit-8 (CCK-8), cell growth curve, colony formation assay, and EdU proliferation assay were applied to evaluate proliferation of hADSCs, cells cycle was investigated by flow cytometry, and adipogenesis was detected by Oil Red O staining and Western blotting. Our results showed that UHRF1 can promote proliferation of hADSCs after overexpression of UHRF1, while proliferation of hADSCs was reduced through downregulation of UHRF1, and UHRF1 can control proliferation of hADSCs through transition from G1-phase to S-phase; besides, we found that UHRF1 negatively regulates adipogenesis of hADSCs via PPAR***γ***. In summary, the results may provide a new insight regarding the role of UHRF1 on regulating proliferation and differentiation of hADSCs.

## 1. Introduction

Obesity may lead to a series of serious metabolism diseases, such as hypertension, diabetes, cardiovascular disease, and dyslipidemia [1]. An increase in adipocytes number (hypertrophy) and size (hyperplasia) may significantly induce obesity [2]; thus deep understanding of the mechanism of adipocyte's proliferation and adipogenesis is of great importance.

Adipose tissue-derived stem cells (ADSCs) possess two significant features, involving multipotential differentiation and self-renewal, and proliferation of ADSCs is complex, while that can be precisely controlled by a variety of physiological processes and regulatory molecules [3–7]. The process of adipogenesis is also regulated by several signaling pathways, such as transforming growth factor beta (TGF*β*), Wnt, glycogen synthase kinase-3*β* (GSK3*β*), and Notch signaling pathway [8, 9]. In addition, it has been reported that the peroxisome proliferator-activated receptor *γ* (PPAR*γ*), cytidine-cytidine-adenosine-adenosine-thymidine (CCAAT)-enhancer-binding protein *α* (C/EBP*α*), and sterol regulatory element-binding proteins (SREBPs) are important nucleus transcription factors, participating in adipogenesis [10].

Overexpression of ubiquitin-like with PHD and RING Finger domains 1 (UHRF1) in a variety of haematological and tumors was noted beforehand, as well as a significant association of its remarkable expression with attenuated expression of a number of tumor susceptibility genes (TSGs). Besides, UHRF1 includes four structural domains: a ubiquitin-like (UBL) domain, a plant homeodomain (PHD) domain, a SRA (SET and RING-associated) domain, and a RING domain [11]. UHRF1 is able to regulate DNA-methylation via different DNA-binding proteins, such as histone H3 lysine 9 (H3K9), histone deacetylase 1 (HDAC1), DNA methyltransferase 1 (DNMT1), proliferating cell nuclear antigen (PCNA), and euchromatic histone-lysine N-methyltransferase 2 (EHMT2)[12, 13]. An another important function of UHRF1 is to promote cell proliferation, which has been extensively reported [14–16].

However, a number of studies indicated that UHRF1 may play different roles in proliferation of different cells. For instance, in tumor cells, the expression of UHRF1 may be easily noted [17], while in some terminal differentiation cells, e.g., UHRF1 is hardly expressed in skeletal muscle cells [18]. At G1/S transition, previous researches demonstrated the efficacy of downregulation of UHRF1 for cell cycle arrest, in which a p53/p21Cip1/WAF1-dependent DNA-damage checkpoint plays a substantial role if that would be activated [19, 20]. UHRF1 inhibitors have possessed precious therapeutic influences in form of being anticancer, in addition to restoration of normal gene expression [21, 22]. However, based on a previous study, UHRF1 can control the self-renewal of HSC via regulation of the cell-division modes epigenetically [23]. Expression of UHRF1 was noted beforehand in early phase of the lineage, while accompanied with other consequences in later phases of survival and neuronal differentiation [24]. Moreover, UHRF1 can colocalize with the maintenance DNMT1 protein throughout S-phase [12].

In this study, we attempted to explore whether UHRF1 can regulate proliferation and differentiation of human ADSCs (hADSCs). Our results demonstrated that UHRF1 could promote proliferation hADSCs after overexpression of UHRF1, whereas proliferation of hADSCs was decreased through downregulation of UHRF1. In addition, we found that UHRF1 negatively regulated adipogenesis of hADSCs via PPAR***γ***.

## 2. Patients and Methods

### 2.1. Patients and Clinical Tissue Specimens

Three male patients with peptic ulcer were recruited in this study. The patients had no acute inflammation, diabetes, malignant tumors, smoking, and mental illness. The abdominal subcutaneous adipose tissues (SATs) were separated from the subjects via a surgical method. This study was approved by the Ethics Committee of The Third Xiangya Hospital of Central South University (Changsha, China). All the subjects signed the written informed consent form.

### 2.2. Isolation, Cultivation, and Differentiation of hADSCs

Here, SAT (0.010 kg) was washed four times with phosphate-buffered saline (PBS), and then, SAT was cut and digested with collagenase I (Sigma-Aldrich, St Louis, MO, USA) at 37°C for 90 min. Next, 10 ml DMEM/F12 (Life Technologies, Carlsbad, CA, USA) was added into centrifuge tube to terminate digestion, and then the medium was filtered by a nylon mesh and was subsequently centrifuged at 150×g for 10 min. After that, the supernatant was gently poured out; 3 ml erythrocyte lysate was added into tube (Beyotime Institute of Biotechnology, Shanghai, China) and centrifuged at 150 g for 10 min; the supernatant was gently poured out again and washed by D-Hank's solution one time and again centrifuged at 150 g for 10 min. The pelleted cells were seeded in DMEM/F12 containing 10% fetal bovine serum (FBS; Life Technologies, Carlsbad, CA, USA). In addition, 4-6 passage cells were used for the next experiments. The specific cell surface markers of hADSCs were detected using a flow cytometer (Muse EasyCyte, Merck Millipore, Germany) with CD73, CD44, CD45, and CD105 (all purchased from eBioscience, Inc., San Diego, CA, USA) and CD90 and CD34 (BioLegend, San Diego, CA, USA). Here, the applied method was according to Wu et al.'s research [25]. Besides, the differentiation protocol of hADSCs was based on our previous study [26].

### 2.3. RNA Extraction and Quantitative Reverse Transcription Polymerase Chain Reaction (RT-qPCR)

Total RNA was extracted by TRIzol reagent (Life Technologies, Carlsbad, CA, USA), and cDNA synthesis was performed with a reverse transcription kit (Promega, Madison, WI, USA). The RT-qPCR was applied by a Mastercycler®ep real-time PCR (Eppendorf, Hamburg, Germany). The relative gene expression was calculated by 2^-ΔΔCT^. These experiments were carried out for three times. Primer sequences used for RT-qPCR are listed in Table 1.

### 2.4. Transfection of hADSCs with Lentivirus

Human lentivirus-UHRF1 (LV-UHRF1) and lentivirus negative control (LV-NC) sequences were constructed by GeneChem Co. Ltd. (Shanghai, China) and transfected into hADSCs according to the protocol. The cells were divided into LV-UHRF1 and LV-NC groups. The expression vector (GV341) contained whole coding sequence of UHRF1. After hADSCs reached confluency of 40-50%, hADSCs were transfected by LV-UHRF1 or LV-NC with 2 mg/ml polybrene (GeneChem Co. Ltd., Shanghai, China) in serum-free medium. After 16 h, the medium was abandoned and replaced with a fresh medium.

### 2.5. Small Interfering RNA (siRNA)

In this phase, hADSCs were seeded at 1 × 10^5^ cells/well and cultured in six-well plates. After 24 h, cells were transfected with 40 nM siRNA-UHRF1 or siRNA-negative control (si-NC). Lipofectamine 3000 was used as transfection reagent (Life Technologies, Carlsbad, CA, USA), and cells were divided into siRNA-UHRF1 group and si-NC group. Three sequences of siRNA-UHRF1 were synthesized, and siRNA-UHRF1 was tested by Western blotting.

### 2.6. Western Blot Analysis

The cells were lysed with radioimmunoprecipitation assay (RIPA) buffer (Sigma-Aldrich, St Louis, MO, USA) and protein concentrations were quantified by bicinchoninic acid (BCA) assay (Beyotime Institute of Biotechnology, Shanghai, China). The proteins were separated by sodium dodecyl sulphate-polyacrylamide gel electrophoresis (SDS-PAGE) and electroblotted onto a polyvinylidene difluoride (PVDF) membrane (Millipore, Billerica, MA, USA). The PVDF membrane was then blocked in 5% skimmed milk and 0.1% Tween-20 at room temperature for 1.5 h and subsequently was incubated in primary antibody overnight at 4°C. The membranes were washed and incubated with horseradish peroxidase (HRP)-conjugated secondary antibodies (Proteintech, Wuhan, China) for 1 h at room temperature and again washed and developed using an enhanced chemiluminescence (ECL) kit (Beyotime Institute of Biotechnology, Shanghai, China). The relative protein expression was analyzed by Quantity One v4.6.2 (Bio-Rad Laboratories, Inc., Hercules, CA, USA). The primary antibodies (UHRF1, Cyclind D1, PCNA, PPAR*γ*, and *β*-actin) were purchased from Proteintech (Wuhan, China).

### 2.7. Cell Proliferation Assay and Cell Growth Curve

The proliferation of hADSCs was assessed by using Cell Counting Kit-8 (CCK-8) (Beyotime Institute of Biotechnology, Shanghai, China) according to the manufacturer's instructions. Briefly, 1 × 10^4^ cells/well were transferred into a 96-well cell culture plate and grew overnight. After 24 h, the cells were transfected with LV-UHRF1, LV-NC, siRNA-UHRF1, or si-NC. After 24, 48, and 72 h, 20 *μ*l CCK-8 was added to each well, and then the plates were incubated for 2 h. Eventually, absorbance was measured at 450 nm with a microplate reader (Bio-Rad Laboratories, Inc., Hercules, CA, USA). The cell growth curve was used by Cell Counting Instrument (Countess II; Thermo Fisher Scientific, Waltham, MA, USA).

### 2.8. Colony Formation Assay

Here, 1,000 cells were seeded in six-well plates and cultured for 10 days. Then, each well was washed with PBS for three times, subsequently fixed with 75% ethanol for 10 min, and stained with 0.1% crystal violet for 30 min. The colonies were observed and counted under a light microscope (Olympus, Tokyo, Japan).

### 2.9. Cell Cycle Analysis

After hADSCs were transfected by siRNA-UHRF1 and LV-UHRF1 for 72 h, respectively, the two groups were harvested and washed with PBS and then fixed by 70% ice-cold ethanol at 4°C overnight. The cells were incubated in PBS with 10 mg/mL RNase and 1 mg/mL propidium iodide (PI; Beyotime Institute of Biotechnology, Shanghai, China) for 1 h at room temperature. The cell cycle was tested using a flow cytometer (Muse EasyCyte, Merck Millipore, Germany) and was analyzed with EasyCyte software according to the standard procedure.

### 2.10. EdU Proliferation Assay

EdU proliferation assay was undertaken by EdU proliferation kit (Beyotime Institute of Biotechnology, Shanghai, China) according to the manufacturer's protocol. Briefly, 1 × 10^4^ cells were seeded in six-well plates; 24 h later, the cells were incubated by 1 ml cell culture medium with 10 *μ*M EdU for 2 h, fixed by 4% paraformaldehyde for 15 min, washed with PBS for three times, incubated at PBS with 0.3% Triton X-100 for 15 min, and then twice washed with PBS. Next, endogenous peroxidase was inactivated by sealing solution for 20 min at room temperature and then stained with DAB working solution (0.1% (w/v) DAB, 0.024% (v/v) H.O., in 0.05 M Tris-HCl, pH 7.6) for 20 min. Nucleus was stained by DAPI (4′,6-diamidino-2-phenylindole) solution. Images were eventually taken by a fluorescence microscope (Olympus, Tokyo, Japan).

### 2.11. Oil Red O Staining

The cells were washed three times with PBS at 37°C, fixed with 4% paraformaldhyde (PFA; Beyotime Institute of Biotechnology, Shanghai, China) for 30 min, washed three times with PBS, and stained with freshly prepared 60% Oil Red O solution (Beyotime Institute of Biotechnology, Shanghai, China) for 20 min at room temperature. The cells were then washed three times with water, and the stained lipid droplets were observed under a light microscope (Olympus, Tokyo, Japan).

### 2.12. Statistical Analysis

The results were presented as mean ± standard deviation (SD). Two groups were compared by the unpaired Student's t-test, and multiple groups were analyzed by one-way analysis of variance (ANOVA). Statistical significance was defined as a P-value < 0.05.

## 3. Results

### 3.1. UHRF1 Regulates Proliferation of hADSCs

**Figure 1:**
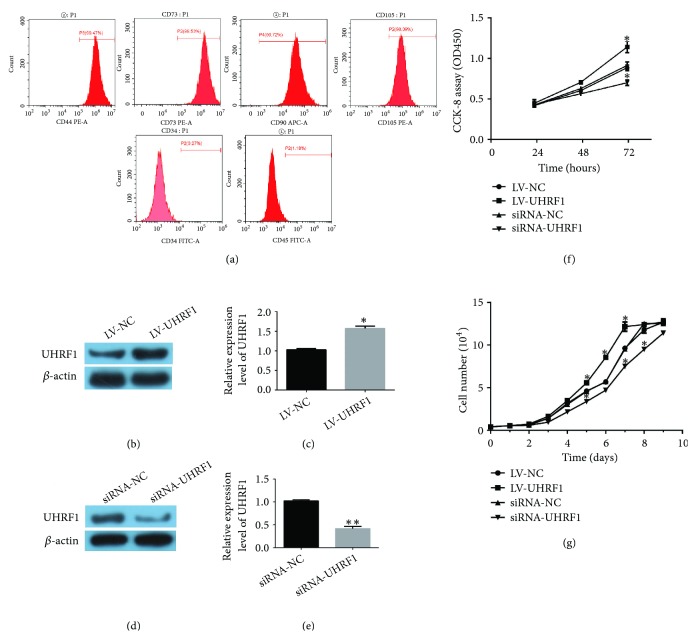


We first detected the identification and characterization of hADSCs, and our results showed that the typical surface marker of mesenchymal stem cells (MSCs) was expressed in hADSCs. Besides, the hADSCs were positive for the mesenchymal markers (CD44, CD73, CD90, and CD105) and were negative for hematopoietic and endothelial markers (CD34 and CD45) (Figure 1(a)). Next, we further explored the expression of UHRF1 after LV-NC, LV-UHRF1, si-NC, and siRNA-UHRF1 were transfected into hADSCs. We found that UHRF1 was significantly upregulated after overexpression of UHRF1 (*∗*P < 0.05 compared with LV-NC group; Figures 1(b) and 1(c)). On the contrary, UHRF1 was significantly upregulated after overexpression of UHRF1 (*∗∗*P < 0.01 compared with siRNA-NC group; Figures 1(d) and 1(e)). To investigate the effects of UHRF1 on proliferation of hADSCs, LV-NC, LV-UHRF1, si-NC, and siRNA-UHRF1 were transfected into hADSCs, respectively. Besides, CCK8 was used to assess proliferation of hADSCs, in which the results showed that the proliferation of LV-UHRF1 group was significantly increased, while that for siRNA-UHRF1 group was notably decreased compared with LV-NC group and si-NC group after cells were transfected for 72 h (*∗*P < 0.05 compared with LV-NC group; Figure 1(f)); next, cell growth curve was further assessed for proliferation of hADSCs; after hADSCs were transfected, the number of cells was daily counted by Cell Counting Instrument (Countess II FL Automated Cell Counter; Invitrogen, Carlsbad, CA, USA), in which the number of cells in LV-UHRF1 group was markedly increased compared with LV-NC group and siRNA-NC group after 5-7 days, while it significantly decreased for siRNA-UHRF1 group in comparison with LV-NC group and si-NC group (*∗*P < 0.05 compared with LV-NC group; Figure 1(g)). The results indicated that overexpression of UHRF1 may promote proliferation of hADSCs, whereas downregulation of UHRF1 may inhibit proliferation of hADSCs.

### 3.2. UHRF1 Accelerates Colony Formation of hADSCs

**Figure 2:**
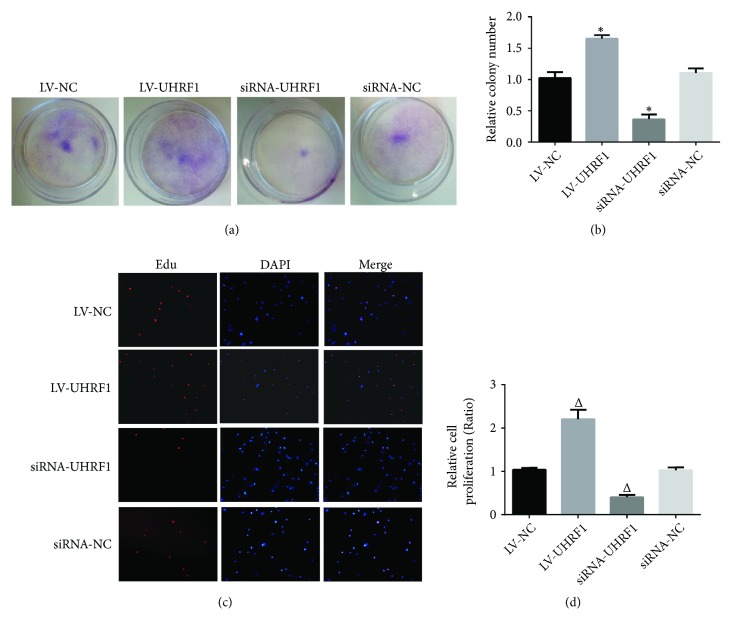


To further explore whether UHRF1 affects colony formation of hADSCs, UHRF1 was upregulated or downregulated in hADSCs; after 10 days, the colony formation of hADSCs was stained with 0.1% crystal violet and observed by a microscope. The findings demonstrated that upregulation of UHRF1 notably promoted colony formation of hADSCs, while downregulation of UHRF1 significantly depressed colony formation of hADSCs (*∗*P < 0.05 compared with LV-NC group; Figures 2(a) and 2(b)). Next, we further used EdU proliferation assay to assess proliferation of hADSCs, in which we found that overexpression of UHRF1 markedly increased proliferation of hADSCs, whereas knockdown of UHRF1 remarkably decreased proliferation of hADSCs (^△^P < 0.05 compared with LV-NC group; Figures 2(c) and 2(d)).

### 3.3. UHRF1 Promotes G1- to S-Phase Transition and Regulates Expression of Cell Cycle-Related Proteins in hADSCs

**Figure 3:**
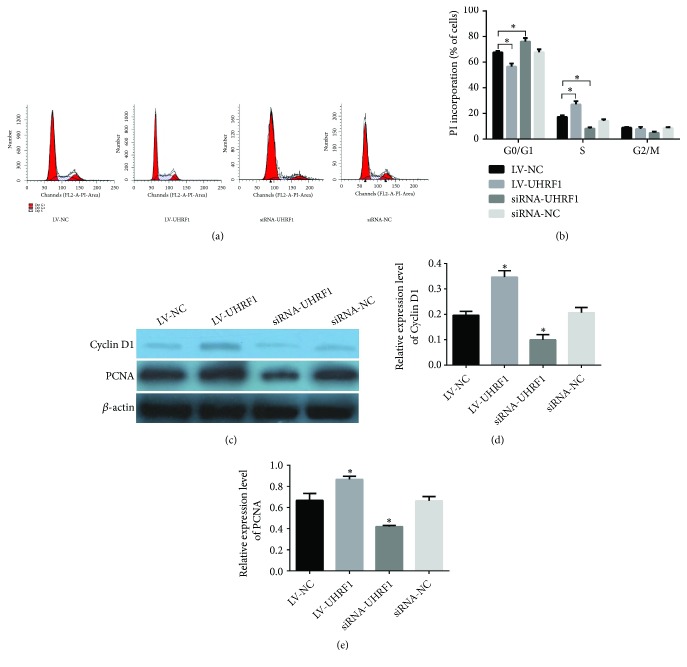


To indicate how UHRF1 affects proliferation of hADSCs, flow cytometry was carried out to investigate the alteration of cell cycle protein expression in hADSCs. The results indicated that G1- to S-phase transition in LV-UHRF1 group was significantly downregulated in comparison with LV-NC group, while proportion of LV-UHRF1 in S-phase was markedly upregulated compared with LV-NC group, and the S-phase was significantly decreased in siRNA-UHRF1 group (*∗*P < 0.05 compared with LV-NC group; Figures 3(a) and 3(b)). Furthermore, the expression level of Cyclind D1 and PCNA was detected by Western blotting, and the mentioned level was markedly increased in LV-UHRF1 group compared with LV-NC group; however, that level was notably decreased in siRNA-UHRF1 group (*∗*P < 0.05 compared with LV-NC group; Figures 3(c), 3(d) and 3(e)). These results indicated that UHRF1 may initiate S-phase through upregulating the expression of cell cycle-related proteins.

### 3.4. UHRF1 Regulates Adipogenesis via PPAR*γ*

**Figure 4:**
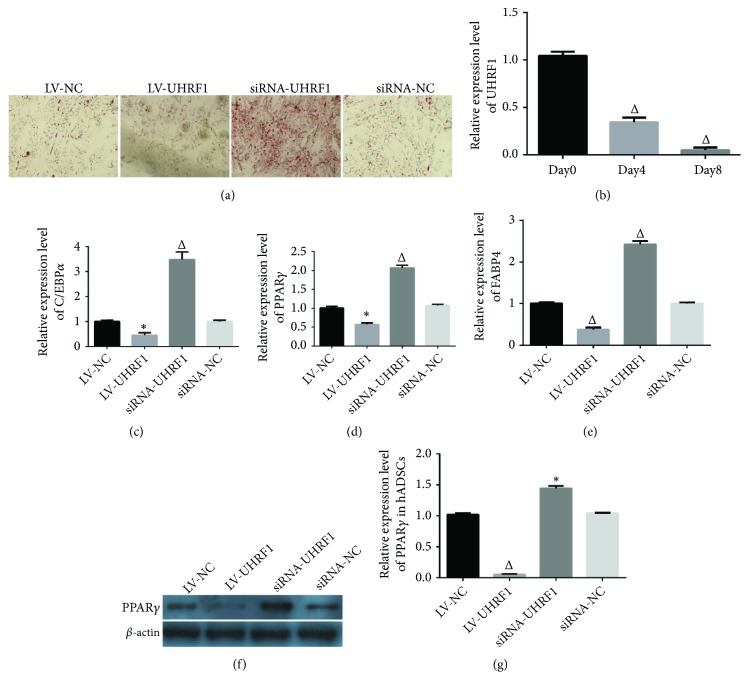


To indicate whether UHRF1 can regulate adipogenesis, the hADSCs were transfected by LV-NC, LV-UHRF1, siRNA-UHRF1, and siRNA-NC, respectively; then those were cultured for 8 consecutive days, and Oil Red staining was undertaken to evaluate cellular lipid droplets in each group. The findings showed that overexpression of UHRF1 could significantly inhibit adipogenesis (Figure 4(a)). It was also revealed that the expression of UHRF1 mRNA was gradually downregulated during adipogenesis (^△^P < 0.01 compared with 0th day; Figure 4(b)). At 8th day, RT-qPCR was carried out to detect the expression of PPAR*γ*, C/EBP*α*, and fatty acid binding protein 4 (FABP4) mRNA in each group. It was disclosed that the expression of PPAR*γ*, C/EBP*α*, and FABP4 mRNA was significantly downregulated in overexpressed UHRF1 group, while it was upregulated in downregulated UHRF1 group (*∗*P < 0.05, ^△^P < 0.01 compared with LV-NC group; Figures 4(c), 4(d) and 4(e)). Next, we analyzed the expression of PPAR*γ* and C/EBP*α* mRNA after LV-NC, LV-UHRF1, siRNA-UHRF1, and siRNA-NC were transfected into hADSCs for 3 days, respectively, and we found that overexpression of UHRF1 could inhibit expression of PPAR*γ*, whereas downregulation of UHRF1 could promote expression of PPAR*γ* (*∗*P < 0.05,^△^P < 0.01 compared with LV-NC group; Figures 4(f) and 4(g)).

## 4. Discussion

In this study, we demonstrated that UHRF1 is a critical factor to regulate proliferation and differentiation of hADSCs. Although a number of previous studies reported that UHRF1 did not affect proliferation in certain stem cells [23, 24]; however, UHRF1 may play a different role in proliferation of hADSCs.

Some studies have shown that UHRF1 plays a major role in proliferation of cells. Besides, UHRF1 has been extensively studied in tumor pathogenesis [27–29], and UHRF1 can maintain methylation status of tumor suppressor genes. Once UHRF1 is upregulated, the expression of those tumor suppressor genes is downregulated, which may cause tumorigenesis [21]. On the other hand, UHRF1 promotes or does not affect proliferation of cells, especially in high proliferation capacity of tumor cells [17], and overexpression of UHRF1 notably actives proliferation, while downregulation of UHRF1 blocks proliferation. However, a number of studies have reported that increase of UHRF1 can block contact inhibition [30, 31]. In addition, UHRF1 cannot affect proliferation and terminal differentiation of certain stem cells [12, 18, 23, 24]. However, no study has indicated whether UHRF1 can affect proliferation and terminal differentiation of hADSCs. Our results showed that UHRF1 can regulate proliferation of hADSCs, and increase or decrease of UHRF1 may enhance or inhibit proliferation of hADSCs.

In order to indicate whether the mechanism of UHRF1 may affect proliferation of hADSCs, we detected cycle changes in hADSCs after overexpression or silencing of UHRF1. The majority of previous studies have illustrated that UHRF1 can regulate proliferation of cells through transition from G1-phase to S-phase, enforce cell cycle from G1/S- to S-phase, in addition to increase cell proliferation [21, 22]. A previous study tested the expression of UHRF1 by immunohistochemistry in specimens of esophageal squamous cell carcinoma (ESCC) patients who treated with radiotherapy, in which it was revealed that UHRF1 was significantly overexpressed in ESCC specimens [32]. The results of the present study showed that UHRF1 controls proliferation of hADSCs through transition from G1-phase to S-phase, which is consistent with those reported previously [19, 20]. At G1 and G2/M phases, we found that expression of novel NP95 was suppressed in normal thymocytes, while it was remarkably expressed in mouse T cell lymphoma cells [33].

To date, no study has indicated whether UHRF1 can affect differentiation of hADSCs. The overexpression or downregulation of UHRF1 was used to explore the role of UHRF1 in adipogenesis, in which our results showed that UHRF1 negatively regulates adipogenesis. It was previously shown that UHRF1 negatively regulates PPAR*γ* and increases proliferation, migration, and clonal formation in colorectal cancer cells lines, and the molecular mechanism revealed that UHRF1 recruits PPAR*γ* promoter and accelerates DNA methylation and repressive histone modification [34]. In the present study, we found that UHRF1 was gradually downregulated during adipogenesis, and also overexpression of UHRF1 might downregulate PPAR*γ* in hADSCs, while downregulation of UHRF1 might increase expression of PPAR*γ*.

In addition, PPAR*γ*, as a nucleus transcription factor, was found to negatively regulate cell proliferation, in which upregulation of PPAR*γ* significantly decreased proliferation in human breast cancer cells or colon cancer cells, [34, 35]. In contrast, reduced expression of PPAR*γ* could increase proliferation in smooth muscle cells (SMCs), and nesfatin-1 could stimulate vascular SMCs (VSMCs) thorough inhibiting PPAR*γ* [36, 37].

Taken together, our results indicated that UHRF1 can promote proliferation of hADSCs and suppress adipogenesis thorough inhibiting PPAR*γ*, and this study may provide a new insight for effective treatment of obesity and related metabolic diseases.

## Figures and Tables

**Table 1:** Primer sequences used for RT-qPCR.

Gene name	primer sequence(5'-3')
UHRF1	5'- GCCATACCCTCTTCGACTACG -3'
	5'- GCCCCAATTCCGTCTCATCC -3'
C/EBP*α*	5'-TGGACAAGAACAGCAACGAG-3'
	5'-TTGTCACTGGTCAGCTCCAG-3'
PPAR*γ*	5'- GAGAAGACTCAGCTCTAC-3'
	5'- CAAGCATGAACTCCATAGTG-3'
FABP4	5'-AGCACCATAACCTTAGATGGGG-3'
	5'- CGTGGAAGTGACGCCTTTCA-3'
GAPDH	5'-GGCTGAGAACGGGAAGCTTGTCAT-3'
	5'-CAGCCTTCTCCATGGTGGTGAAGA-3'

## Data Availability

All data can be presented by the corresponding author upon request.
